# Altered Right Ventricular Kinetic Energy Work Density and Viscous Energy Dissipation in Patients with Pulmonary Arterial Hypertension: A Pilot Study Using 4D Flow MRI

**DOI:** 10.1371/journal.pone.0138365

**Published:** 2015-09-29

**Authors:** Q. Joyce Han, Walter R. T. Witschey, Christopher M. Fang-Yen, Jeffrey S. Arkles, Alex J. Barker, Paul R. Forfia, Yuchi Han

**Affiliations:** 1 Cardiovascular Division, University of Pennsylvania, Philadelphia, PA, United States of America; 2 Department of Radiology, University of Pennsylvania, Philadelphia, PA, United States of America; 3 Department of Bioengineering, University of Pennsylvania, Philadelphia, PA, United States of America; 4 Department of Radiology, Northwestern University, Chicago, IL, United States of America; 5 Cardiovascular Division, Temple University, Philadelphia, PA, United States of America; Nippon Medical School Graduate School of Medicine, JAPAN

## Abstract

**Introduction:**

Right ventricular (RV) function has increasingly being recognized as an important predictor for morbidity and mortality in patients with pulmonary arterial hypertension (PAH). The increased RV after-load increase RV work in PAH. We used time-resolved 3D phase contrast MRI (4D flow MRI) to derive RV kinetic energy (KE) work density and energy loss in the pulmonary artery (PA) to better characterize RV work in PAH patients.

**Methods:**

4D flow and standard cardiac cine images were obtained in ten functional class I/II patients with PAH and nine healthy subjects. For each individual, we calculated the RV KE work density and the amount of viscous dissipation in the PA.

**Results:**

PAH patients had alterations in flow patterns in both the RV and the PA compared to healthy subjects. PAH subjects had significantly higher RV KE work density than healthy subjects (94.7±33.7 mJ/mL vs. 61.7±14.8 mJ/mL, p = 0.007) as well as a much greater percent PA energy loss (21.1±6.4% vs. 2.2±1.3%, p = 0.0001) throughout the cardiac cycle. RV KE work density and percent PA energy loss had mild and moderate correlations with RV ejection fraction.

**Conclusion:**

This study has quantified two kinetic energy metrics to assess RV function using 4D flow. RV KE work density and PA viscous energy loss not only distinguished healthy subjects from patients, but also provided distinction amongst PAH patients. These metrics hold promise as imaging markers for RV function.

## Introduction

The complex geometry and retrosternal location of the right ventricle (RV) confound accurate RV assessment by echocardiography. As a result, cine MRI is the reference standard for RV assessment due to its superior three-dimensional (3D) anatomy delineation and volume calculations [1–4]. RV structural changes occur in response to pulmonary vascular diseases such as pulmonary arterial hypertension (PAH) and may improve in response to therapy [5–7]. However, MRI structural parameters may lag behind clinical improvement [7], and there is a need for the development of more sensitive and early imaging markers. Given that alterations of intracardiac and great vessel flow may precede RV structural changes, the dynamic assessment of complex intracardiac flow may be a promising tool to potentially improve prognosis and assessment of therapeutic efficacy. Nonetheless, a comprehensive approach to visualizing blood flow has remained a challenge due to the limited options for imaging RV flow. Recently, time-resolved 3D phase-contrast MRI (4D flow- as velocity is encoded in 3 dimensions and the time dimension over the cardiac cycle is the 4^th^ dimension) [8–12] has provided insights into a number of complex flow patterns in congenital heart disease [13, 14], aortic valve disease [15], the disease of the aorta [16–18], and pulmonary hypertension[19–21].

Efforts have been made to develop quantitative parameters using 4D flow to study the heart and the great vessels. For example, Fredrikson et al. calculated kinetic energy of each of the four intracardiac flow components: direct flow, retained inflow, delayed ejection flow, and residual volume [11]. In the great vessels, parameters such as total flow, peak systolic velocity, and wall shear stress have shown excellent scan-rescan, inter-observer, and intra-observer reproducibility [13]. In addition, Barker et al. compared various flow structures as well as wall shear stress measurements measured using Cartesian- and radial-based 4D flow MRI acquisitions, and found similar results.[21] The duration of vortex formation in the main pulmonary artery (PA) was shown to allow accurate estimation of PA pressure and diagnose patients with pulmonary hypertension. [12, 20].

PA vortex formation increases fluid-mechanical losses and further increase cardiac after load. Thus, understanding the impact of flow abnormalities and their role in the loss of useful cardiac stroke work is important to elucidate the overall RV workload. This is especially relevant in the case of PAH, which often culminates in RV failure.

We propose to use 4D flow MRI to study kinetic energy efficiency of the RV and viscous energy loss in the PA of subjects with PAH. These two parameters can be calculated from the velocity data obtained in 4D flow and potentially can be used clinically to assess patients.

## Materials and Methods

### Study Population

Ten PAH subjects were recruited from the pulmonary hypertension clinic under the care of one clinician. All were diagnosed based on mean pulmonary pressure ≥ 25 mmHg measured by right heart catheterization [22]. Nine subjects were designated World Health Organization (WHO) Group 1 patients, and they were on at least one PAH-specific medication. One subject, who was Group 4 (chronic thromboembolic pulmonary hypertension) prior to surgery, was not on any PAH-specific medication. PAH functional classes were accessed using the WHO class system, on the dates of their MRIs. Five patients, who had mild limitation in daily activities, were in functional class II and five patients, who had no limitation in daily activities, were in functional class I. Healthy subjects were recruited from the community and were free of cardiopulmonary disease and were not on any prescription medication. The study was approved by the local institutional review board and written consents were obtained from all subjects.

### MRI Data Acquisition

The study population underwent cardiac MRI exams on a 1.5T Siemens scanner (Avanto, Siemens Health Systems, Germany). 2D cine short axis and long axis slices were acquired. No contrast agent was used during the scans. Cine data were obtained using steady state free procession imaging with the following parameter: TE = 1.1–1.3 ms, TR = 2.2–2.6 ms, matrix = 144–192 x 192, field-of-view = 240–320 mm x 240–400 mm, BW = 930 Hz/pixel, phases = 30, slices = 11–14, slice thickness = 8 mm, gap = 2 mm. Temporal resolution = 29.8 ± 7.0ms. The average breath-hold duration was 12±3s.

The 4D flow data were acquired using a prospective ECG-gated 3D cine PC-MRI sequence covering the entire RA, RV, and the main pulmonary artery. Respiratory navigator gating were used to minimize motion artifacts. The scan parameters were: VENC = 125cm/s, flip angle = 8 degrees, voxel size = 2.5 x 2.5 x 2.5 mm^3^, bandwidth = 400Hz, and slab thickness of 60–70 mm. Acquisition time was 17 to 25 minutes. Following the acquisition, the flow data were reconstructed into 13 to 21 time frames.

### Cine Image Analysis

Cine MRI Images were imported into Argus Viewer (Siemens Health Care, Germany) and the endocardial contours were manually traced in diastole and systole for both LV and RV by an experienced observer. RV mass was obtained in end-systole by drawing epicardial contours on the RV, excluding the septum.

### 4D Flow Image Analysis

Background error removal (noise correction, anti-aliasing, and eddy-current correction) was performed using in-house software [23] in MATLAB (Mathworks, Natick, MA). Pre-processed data were saved in a file format compatible with visualization software EnSight (CEI Inc, Apex, NC) for subsequent pathline visualization and initial parts of kinetic energy computations. The end-systolic time frame was determined as the time point at which the flow through the pulmonic valve is at a minimum. End-diastolic time was taken to be the last time frame. Two circular planes representing the tricuspid and pulmonic valves were drawn using geometry tools in EnSight. The planes were then masked with velocity thresholds to create segmentations that resemble the cross sectional areas, taking into account the change in diameter at different points of the cardiac cycle. In order to minimize the interference of valve movements in the measurements, both planes were placed slightly below the valves (inside of the RV) during the entire time periods of interest, using the cine right ventricular outflow track (RVOT) images as a reference.

### Flow Visualization and Analysis

#### RV kinetic work density

Pathlines were emitted from the two planes from the first time frame to end-systole, tracing both forward and backward in time, encompassing a complete cardiac cycle. Each pathline is considered to represent a volume of blood corresponding to the voxel size. Flow through the tricuspid valve (TV) and pulmonic valve (PV) were calculated as *Q* = ∫_*cardiac cycle*_∫_*valve*_(***v*·*n***) *ds dt*, where ***v*** is the velocity vector at the plane and ***n*** is the normal vector to the plane representing the TV or PV.

Kinetic energy through the TV and PV was calculated as

$$KE=\int_{\begin{array}{c} systole\\ or\;diastole \end{array}}\int_{valve}\frac{1}{2}\rho v^{2}(v\cdot n)dS\;dt,$$ (1)

where *dS* is the differential area element the area of the valve, ***v*** is velocity of each blood particle through the valve, and *ρ*, the blood density, is taken to be 1.06 x 10^3^ kg/m^3^. Total kinetic energy input through tricuspid and pulmonic valves were calculated using Eq 1 with diastolic and systolic durations as the integration limits. The integral was approximated using a trapezoidal Riemann sum [24].

To assess the minimum kinetic energy of the heart as a system in equilibrium, we arrive at the following relation

$$RV\;KE\;work=KE_{out}-KE_{in}-KE_{PR}+KE_{TR}$$ (2)

where KE_in_ and KE_out_ correspond to the kinetic energy coming in through the tricuspid valve and out of the pulmonic valve, respectively; KE_PR_ and KE_TR_ denote the amount of kinetic energy associated with pulmonic regurgitation and tricuspid regurgitation. Tricuspid regurgitation velocity was not measured in this study because of high TR velocity. Assume KE_TR_ ≥ 0, the lower bound of kinetic work becomes KE_out_−KE_in_−KE_PR_ (KE_out_, KE_in_, and KE_PR_ were calculated in EnSight). RV KE work density is then defined as the minimum amount of kinetic work required per stroke volume (SV) in a single cardiac cycle:

$$RV\;KE\;work\;density=\frac{RV\;KE\;work}{SV}$$ (3a)

Thus, the lower bound of RV KE work density is established as:

$$RV\;KE\;work\;density\;\geq \frac{KE_{out}\;–\;KE_{in}\;–\;KE_{PR}}{SV}.$$ (3b)

#### PA viscous energy dissipation

We also analyzed the energy profile in the PA by assessing the irreversible loss of mechanical energy. Viscous dissipation, describing the conversion of mechanical energy into thermal energy per unit volume, was calculated as:

$$\mu \phi_{v}=\frac{1}{2}\mu \sum_{i}\sum_{j}{\left[\left(\frac{\partial v_{i}}{\partial x_{j}}+\frac{\partial v_{j}}{\partial x_{i}}\right)-\frac{2}{3}\left(\nabla \cdot v\right)\delta_{ij}\right]}^{2},$$ (4)

where *μ* is the blood viscosity, *i* and *j* denotes the principal directions *x*, *y* and *z*, and *δ*_*ij*_ is the Kronecker delta [25]. The viscous energy loss rate [26, 27] can then be calculated as

$${E^{\prime }}_{loss}=\int_{Vol}\mu \phi_{v}dV.$$ (5)

The main PA was segmented by tracing regions of interest inside the vessel in all slices containing the vessel. The energy loss rate throughout the cardiac cycle was subsequently calculated using the Eq 5 in the segmented main PA, through numerical spatial derivatives. Integrating E’_loss_PA_ over the cardiac cycle gives us the total amount of kinetic energy loss in the main PA, denoted by E_loss_PA_. The percentage of energy loss in the total KE output can thus be calculated by the ratio of totally dissipated energy and total KE output: E_loss_PA_/KE_out_.

The initial observer repeated the complete series of placing the analysis planes, segmentations, and calculations for three randomly chosen subjects (one health subject and two PAH patients). There was an interval of at least two weeks between the analyses. The results from the respective observations were analyzed using intraclass correlation (ICC).

### Statistical Analysis

Measurements of energy and work density are expressed as mean ± standard deviation, unless stated otherwise. Fisher’s exact test was used to test the significance of differences between the two groups of subjects. Linear regression analysis was performed to test the significance of the correlation between various RV measurements. Results with a p<0.05 were considered statistically significant.

## Results

The demographics, relevant medications, and functional class of the ten PAH patients are listed in Table 1. Most patients had no recent pulmonary arterial pressure information by catheterization or echocardiography. The median time interval between right heart catheterization and MRI for the ten patients was 675.5 days with an interquartile range of [152.0, 1294.3] days. The RV functional parameters of the healthy and PAH subjects are presented in Table 2. No significant differences exist in age or gender. In PAH patients, the LV ejection fraction ranged between 53 to 72%, and the RV ejection fraction ranged between 40 and 54%. This is a group of relatively healthy PAH patients.

**Table 1:** PAH subject clinical characteristics and RV function.

Subjects	Age	Sex	ERA	PDE5	CCB	PGI_2_ analogue	Functional Class	RVEF (%)
PAH01	42	F	-	sildenafil 20 mg	nifedipine 30mg	-	II	50
PAH02	29	F	bosentan 125 mg	tadalafil 20 mg	amlodipine 5 mg	-	II	53
PAH03	53	F	ambrisentan 10 mg	-	amlodipine 2.5 mg	-	II	54
PAH04	48	M	ambrisentan 10 mg	tadalafil 20 mg	-	-	I	40
PAH05	64	F	bosentan 125 mg	sildenafil 20 mg	-	iloprost 20mcg/ml	I	47
PAH06	26	F	bosentan 125 mg	sildenafil 20 mg	-	-	I	42
PAH07	29	F	bosentan 62.5 mg	sildenafil 20 mg	-	-	I	40
PAH08	20	F	ambrisentan 10 mg	sildenafil 20 mg	-	-	I	40
PAH09*	27	F	-	-	-	-	II	41
PAH10	38	M	-	sildenafil 20 mg	-	-	II	45

Abbreviations: ERA = Endothelin receptor antagonists. PDE-5 = Phosphodiesterase Type 5 inhibitor. CCB = Calcium channel blocker. PGI_2_ = prostacyclin. All medications are taken in the usual dosing schedule. PAH09 was not on pulmonary hypertension specific medication due to chronic thromboembolic pulmonary hypertension before surgery.

**Table 2:** Subject RV Functional Characteristics.

	PAH (n = 10)	Control (n = 9)	p value
Gender (male, (%))	2 (20)	3 (33.3)	0.27
Age (years)	48±14	30±13	0.14
BSA (m^2^)	1.9±0.37	1.70 ±0.21	0.85
RVEDV (ml)	181.8±73.9	173.2±33.3	0.38
RVEDVI (ml/m^2^)	96.4 ±26.5	99.9 ±15.8	0.32
RVESV (ml)	114.5 ±48.1	82.5±23.3	0.23
RVSV (ml)	80.3 ±29.1	93.1±17.7	0.15
RVEF (%)	45.2 ±5.5	52.9 ±6.2	0.01
RVM (g)	53.7±22.0	54.3 ±36.5	0.67
RVMI (g/m^2^)	31.1±11.3	28.5±17.4	0.29

Abbreviations: BSA = body surface area. RVEDV = right ventricular end-diastolic volume. RVEDVI = right ventricular end-diastolic volume indexed to BSA. RVESV = right ventricular end-systolic volume. RVSV = right ventricular stroke volume. RVEF = right ventricular ejection fraction. RVM = right ventricular mass. RVMI = right ventricular mass indexed to BSA.

**Fig 1:**
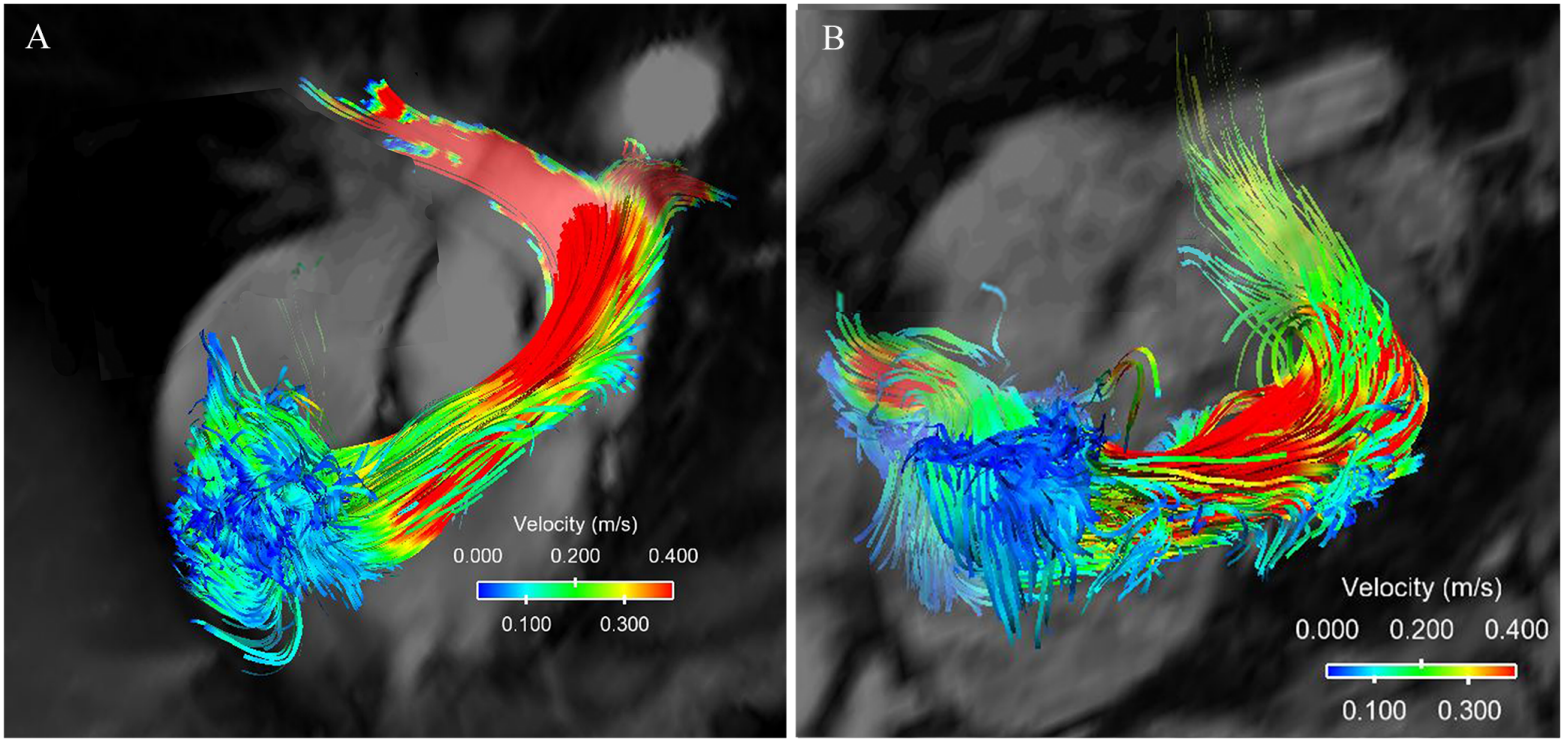
4D flow patterns. (A) Flow pattern of a healthy subject in the right ventricle and the pulmolnary artery. The flow is laminar and is of relatively high velocity. (B) Flow pattern of a PAH patient with severe PA dilation where the vortices form in the PA and the flow velocities are lower.

Pathline visualizations of a healthy subject and a PAH subject are shown in Fig 1, and the respective videos capturing the flow patterns over a complete cardiac cycle are available in the supporting materials (S1 and S2 Videos). No significant difference was visualized pathlines between class I and class II patients. The PAH patient included in Fig 1 had the most representative vortices, and was a class I patient. Healthy subjects have two similarly sized vortices underneath the tricuspid valve during diastole with consistent orientation, size and location in the RV. PAH subjects have much more complex flow features in the RV, with asymmetric vortices, and/or additional vortices. Healthy subjects show a laminar flow pattern inside the PA. PAH subjects with dilated PA show clear helical flow in the PA with vortices persist through systole and dissipate during diastole.

**Fig 2:**
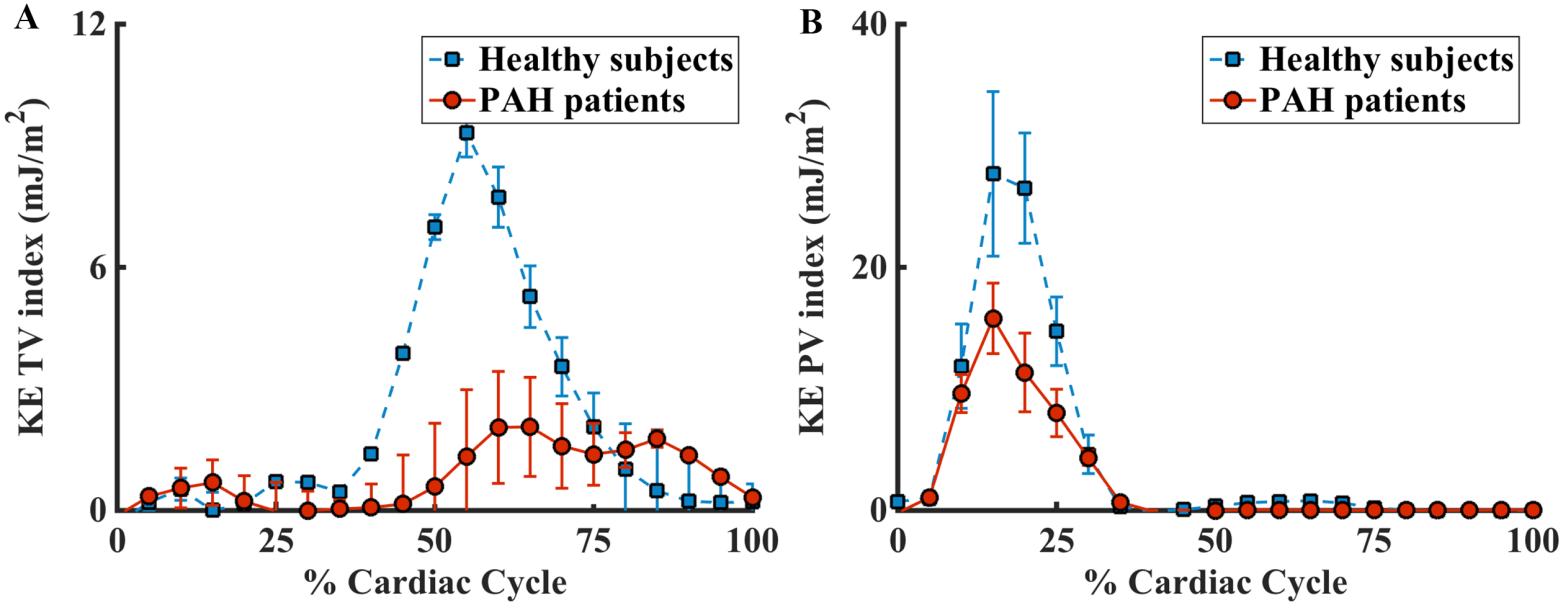
Comparison of the in flow kinetic energy and outflow kinetic energy in healthy subjects and PAH subjects. (A). Normalized KE_in_ through the tricupsid valve. (B). Normalized KE_out_ through the pulmonic valve.

**Fig 3:**
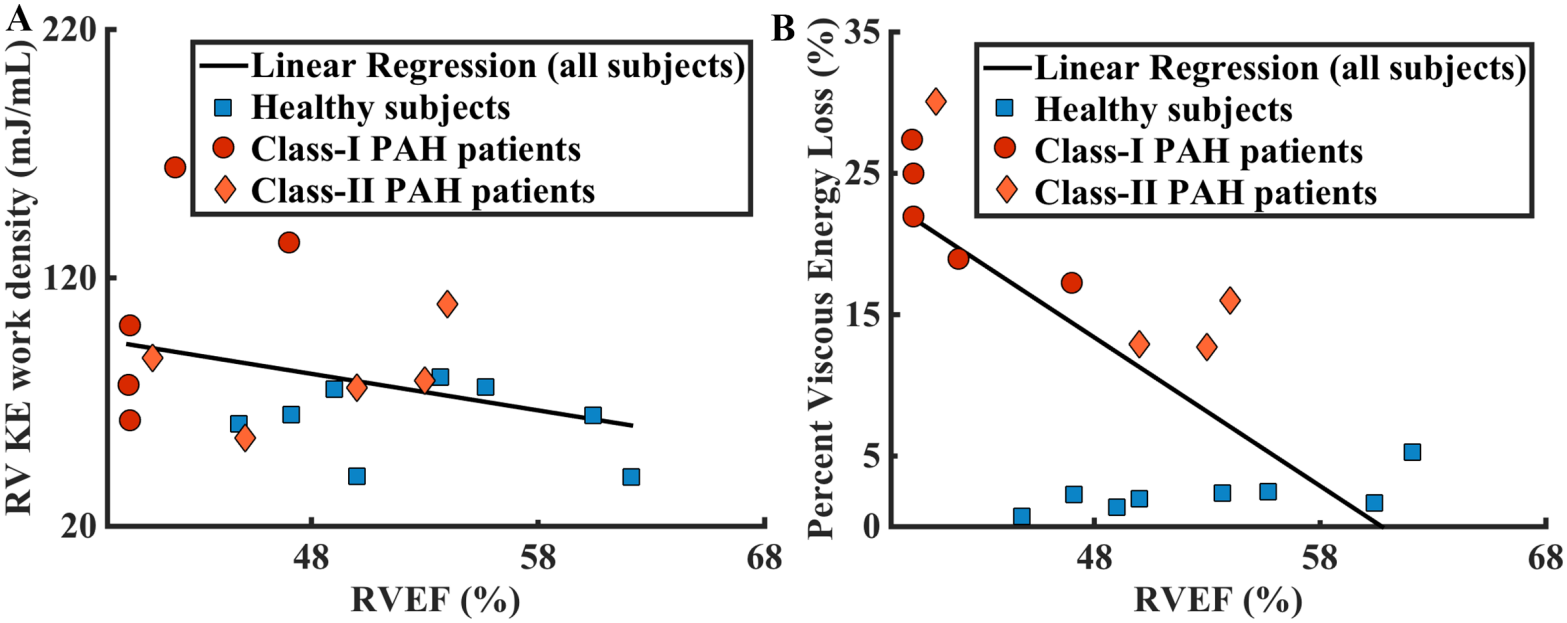
Comparison of kinetic energy density and viscous energy loss in PAH and healthy subjects. A. Healthy subjects showed significantly lower RV KE density than PAH patients (61.7±14.8 mJ/mL vs. 94.7±33.7 mJ/mL, p = 0.007). Linear regression of RV KE density against RVEF for both populations gives result: y = -1.47x + 152.00, R² = 0.11. B. Healthy subjects showed significantly lower percent viscous energy loss than PAH patients (2.2±1.3% vs. 21.1±6.4%, p = 0.0001). Linear regression of percent viscous energy loss against RVEF for both populations gives result: y = -1.05x + 63.82, R² = 0.41.

Comparison of normalized kinetic energy between healthy subjects and PAH patients through the tricuspid (KE_in_) and pulmonic valves (KE_out_) is shown in Fig 2. Higher KE entered into the RV and also left the RV in healthy subjects compared to PAH subjects. Healthy subjects showed significantly lower KE work density than PAH subjects (61.7±14.8 mJ/mL vs. 94.7±33.7 mJ/mL, p = 0.007). RV KE work density did not correlate with age, sex, or functional class, but negatively correlated with body surface area (Table 3). It did not correlate with any of the RV functional measurements except for RVSV and RVEF, where moderate and mild correlations are found (Fig 3A). Note that RV KE work density is dependent on RVSV, by definition (Eqs 3a and 3b). Lastly, RV KE work density did not correlate mean PA flow velocity.

**Table 3:** Multivariate analysis of kinetic work density and percent viscous energy loss to demographic and RV parameters.

	P value	
	Kinetic work density	Percent viscous energy loss
Gender (male, (%))	0.21	0.84
Age (years)	0.63	0.59
BSA (m^2^)	0.03	0.2
WHO class	0.55	0.67
RVEDV (ml)	0.52	0.16
RVESV (ml)	0.85	0.11
RVSV (ml)	0.02	0.76
RVEF (%)	0.18	0.002
RVM (g)	0.8	0.97
Mean PA flow velocity (m/s)	0.53	0.32

Abbreviations: BSA = body surface area. RVEDV = right ventricular end-diastolic volume. RVEDVI = right ventricular end-diastolic volume indexed to BSA. RVESV = right ventricular end-systolic volume. RVSV = right ventricular stroke volume. RVEF = right ventricular ejection fraction. RVM = right ventricular mass. RVMI = right ventricular mass indexed to BSA.

**Fig 4:**
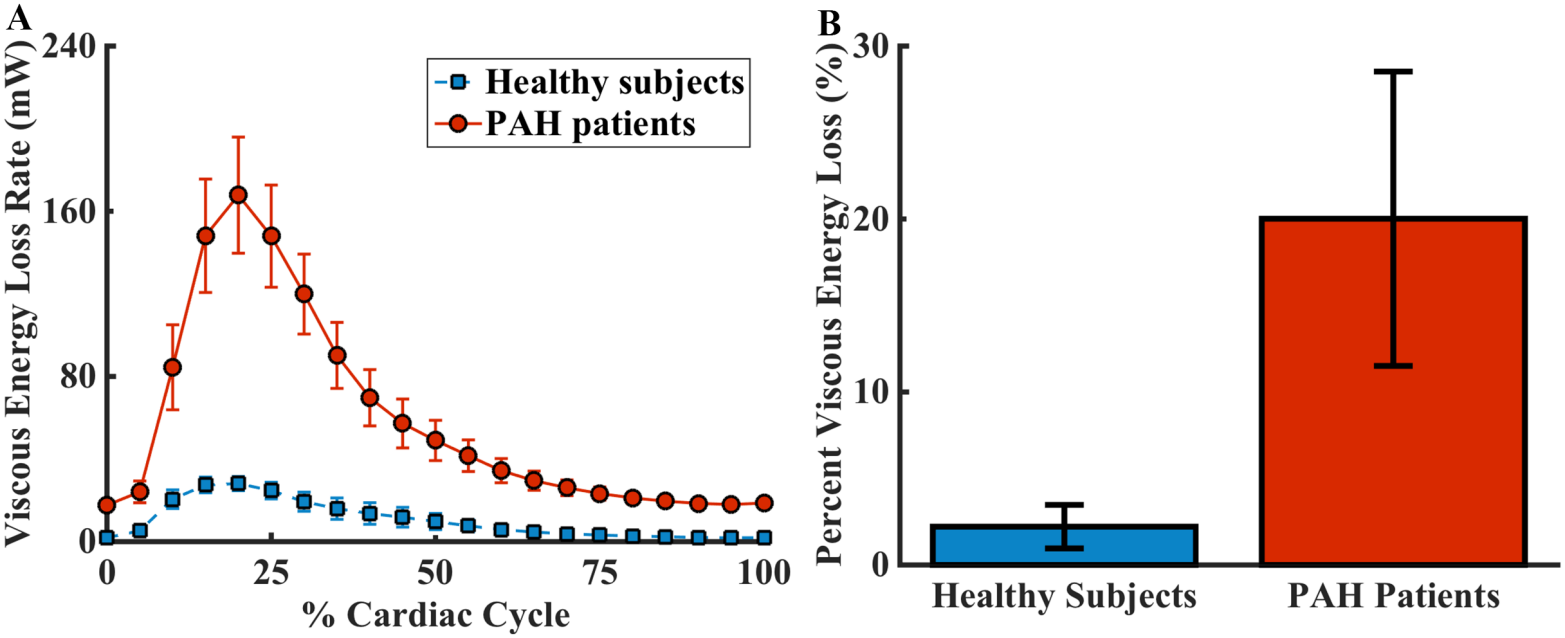
Comparing viscous energy loss in healthy subjects and patients with pulmonary arterial hypertension. (A) The amount of viscous energy loss is higher in patients throughout the cardiac cycle. (B) The percent viscous energy loss is significantly greater in PAH patients than in healthy subjects (21.1±6.4% vs. 2.2±1.3%, p = 0.0001).

The total amount of viscous energy loss in the MPA was higher in patients during all phases of the cardiac cycle, shown in Fig 4A. The percentage of kinetic energy loss in the total kinetic energy output in PAH subjects is significantly greater than that in healthy subjects (121.1±6.4% vs. 2.2±1.3%, p = 0.0001) as shown in Fig 4B. Percent viscous energy loss was not correlated with any of the demographic or clinical parameters (Table 3), but it was moderately correlated with RVEF (Fig 3B).

**Fig 5:**
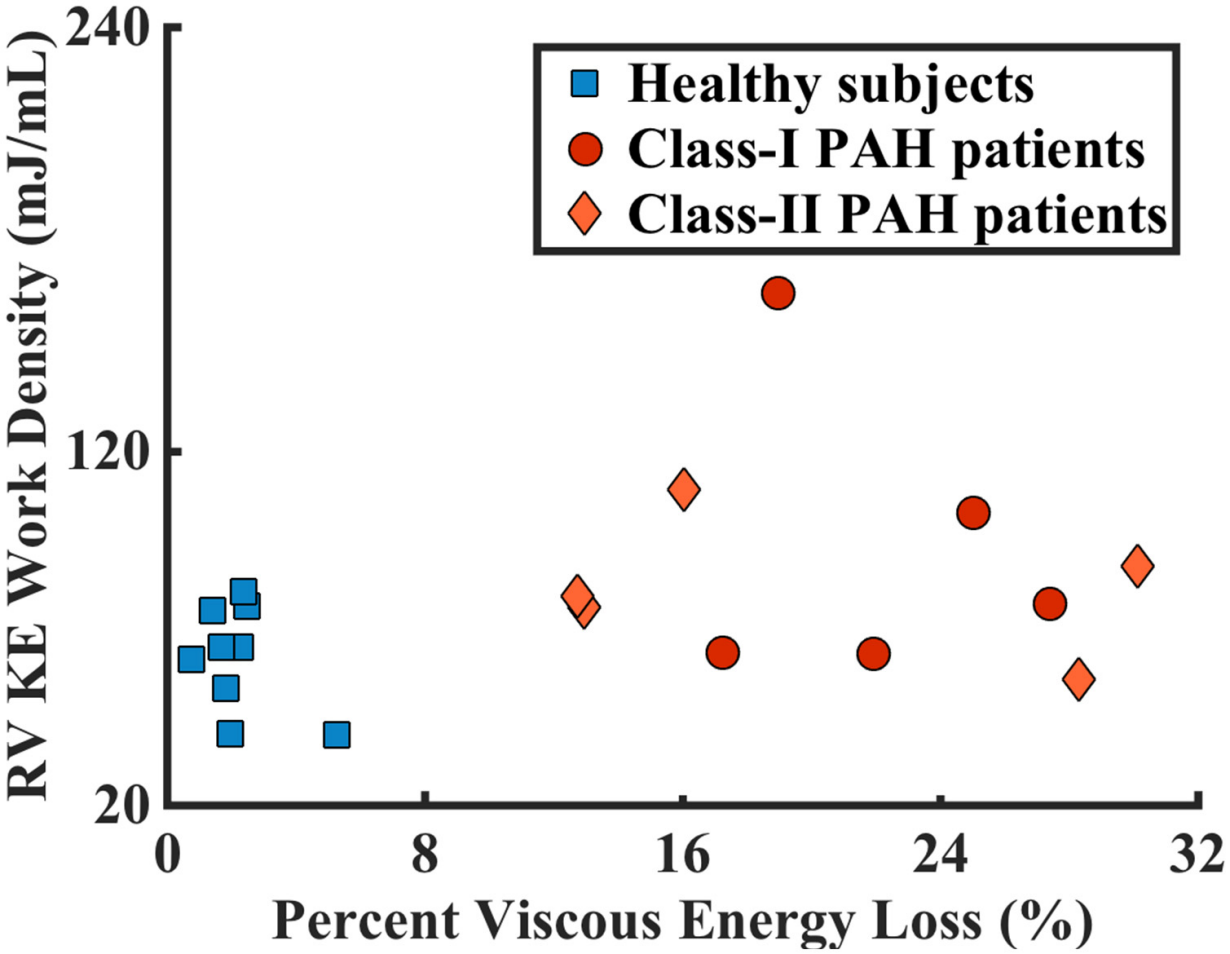
RV KE density and percent viscous energy loss together separated the PAH subjects from the healthy controls. In addition, PAH patients within the same functional classes had a wide distribution.

Plotting the percent viscous energy loss against the RV KE work density clearly separates healthy subjects from the PAH patients (Fig 5). Healthy subjects congregate in the region with low work density and low percent viscous energy loss, whereas the PAH patients were widely distributed on the graph. Regressing work density against percent viscous energy loss for the whole study population showed no correlation (p = 0.10); the congregated and scattered distribution of the two populations also do not show correlations between these two parameters (p = 0.26 for healthy subjects and p = 0.24 for PAH patients).

The consistency of agreement of KE work density and percent viscous energy loss in the randomly chosen subjects was high, 0.990 and 0.995 respectively, based on ICC analysis.

## Discussion

Studies have shown the importance of RV functional measurements in determining PAH patients’ prognosis and response to treatments [5, 28, 29]. MRI has a increasing role in assessing PAH patients [30]. We observed a significant increase in RV KE work density and a significant increase in percent viscous energy loss in the PA in PAH patients compared to normal subjects. These two metrics not only distinguished healthy subjects from patients, but also provided distinction amongst PAH patients, which might provide new insight over the conventional MRI parameters of the RV.

In previous 4D flow studies, ventricular flow has been categorized into four components and each flow component has been characterized in normal subjects [11]. It is estimated that 44 ± 6% of flow from normal subjects is direct flow, that is flow that comes in and goes out in the same cardiac cycle [11]. This quantification requires detailed segmentations of the RV and intensive computation, which is not easily feasible for larger number of patients. We took a different approach for energy estimation. We considered the RV as a system under steady state conditions during which the volume of the ventricle stays constant from one cardiac cycle to the next, and estimated kinetic energy input and output of the system over a complete cardiac cycle based on the first law of thermodynamics. Sources of RV KE input include flow from the right atrium through the tricuspid valve and pulmonic regurgitation, while output includes output through the pulmonic valve and tricuspid regurgitation. Tricuspid regurgitation was not measured due to the high velocity encoding range that is needed to accurately capture the regurgitant jets. Part of the work done by the RV also includes the movement of the RV end systolic volume, which we did not account for in this study. Therefore, we estimated the lower bound of RV work. Despite these omissions for RV work using simplified estimations, lower bound of RV KE work density may still be helpful to assess the energetic work of the RV. Direct flow is visually diminished in PAH subjects in our cohort, which we suspect contribute to the higher RV KE work density.

Blood is a viscous fluid and viscosity is an intrinsic property of a fluid that gives rise to the development of viscous shear stresses inside the flow [31]. Energy dissipation due to viscous stresses transforms mechanical energy in fluids to thermal energy. Vortices are common intracardiac flow features. Vortices developed in the cardiovascular system play fundamental roles in the normal physiology, but the formation of unnatural vortices may alter the momentum transfer in the blood flow and increase energy dissipation [31]. We observed diastolic vortex formation pattern in the RV in healthy subjects is similar with the observed LV filling pattern observed by others [32]. The diastolic vortices are altered in some PAH subjects with additional vortices formed toward the apex of the RV or with asymmetrical vortices (Fig 1B), which suggest abnormal energy dissipation.

In PAH and other high after-load pulmonary hypertensive conditions, the markedly altered PA flow has long been recognized by Doppler echocardiography [33]. The Doppler ‘notching’ relates to the pulmonary artery flow deceleration, as a result of early arrival of reflected waves from high pulmonary artery impedance [34, 35]. This abnormal flow pattern is associated with greater degrees of RV dysfunction and is not present in pulmonary hypertension conditions where the pulmonary vascular resistance is relatively normal. The vortices formed in the main pulmonary artery has been studied in pulmonary hypertension by 4D flow, and are mostly found to be pathological and the persistence of it is related to elevated pulmonary arterial pressure [12, 19].

Some recent studies have attempted to calculate energy loss using 4D flow in different disease states. Barker et al. studied viscous energy loss in the thoracic and ascending aorta at peak systole, and found that to be significantly elevated in patients with dilated aortas as well as in cases of aortic stenosis [27]. Lee et al. studied the rates of pressure-flow and KE transferred between the branch PA and the MPA, as well as flow resistance due to MPA bifurcation, also relations derived from the Navier—Stokes equations. They showed that patients with congenital heart disease had considerably increased energy loss in the branch PAs compared to normal subject with normal PA physiology [36]. Besides energy loss, a modification in the 4D flow technique with asymmetrical 4-point motion encoding has allowed measurement of irreversible pressure loss through characterization of turbulent KE (34).

In our study, percent viscous energy loss in the PA is small in healthy subjects, but is almost an order of magnitude greater in PAH subjects. The increase in PA energy loss in the PAH subjects due to the vortex formation results in the decrease in mechanical energy available for the system, thus leads to elevated RV kinetic energy load. Note that the estimation was performed only in the MPA, a rather small portion of the overall pulmonary system. Thus, the actual loss is expected to be larger than calculated in our study.

Combining lower bound of RV kinetic energy work density and lower bound of percent viscous energy dissipation measurements, we observe a clear differentiation among PAH subjects who are clinically in the same functional class, as shown in Fig 5. This is especially relevant because the RV’s adaptation to the increased after-load is a major determinant of a PAH patient’s functional capacity and survival [6]. These two metrics lead us to hypothesize that the patient with the highest RV kinetic energy work density and the highest viscous energy dissipation might not do well over time and might require escalation of therapy sooner. The scatter plot of these two parameters showed congregated distribution in the healthy population and dispersed distribution in the PAH population. Factors such as the geometry of RV, the complex flow movements inside the RV, energy loss through viscous dissipation, and many others, may contribute different degrees to the increase of work density in PAH patients; thus work density does not directly correlate with any one of these factors alone.

There are limitations in this study in addition to a small sample size. Due to the large data set, the time frames taken did not cover the complete cardiac cycle for a few bradycardiac healthy subjects, which were compensated by extending the existing data to a complete cycle using linear interpolating techniques. Thus, the actual KE_In_ might potentially be greater for these healthy subjects and the actual KE work density is consequently lower. The median time of MRI is nearly two years after RHC, thus we weren’t able to reliably investigate the correlation between hemodynamic data and our analysis. The functional classes used in these analyses were accessed at the time of the MRI. Three patients have comorbid diseases. One patient had systemic sclerosis, but was still working full time; one patient has a restrictive membranous VSD; and one has a secundum ASD that was closed 8 months prior to imaging. With these small numbers, we are unable to assess the impact of these conditions on the results of our study.

In summary, we have quantified two kinetic energy metrics to assess RV function, namely, the lower bound of kinetic energy work density of the RV and lower bound of the percent viscous energy dissipation in the PA, which are solely based on imaging blood volume and velocity. Our work has introduced a quantitative framework and metrics to analyze large amount of data from 4D flow imaging. Our next steps are to evaluate the clinical relevance of these metrics in longer follow-ups and larger patient population. We expect that these measurements will enable a more sensitive assessment of the RV function and its response to treatment, as compared to conventional MRI volumetric measurements.

## Supporting Information

S1 Video Pathline visualizations of a healthy subject over the complete cardiac cycle. (MOV)

S2 Video Pathline visualization of a PAH subject over the complete cardiac cycle. (MOV)
